# Use of Capsaicin to Treat Pain: Mechanistic and Therapeutic Considerations

**DOI:** 10.3390/ph9040066

**Published:** 2016-11-01

**Authors:** Man-Kyo Chung, James N. Campbell

**Affiliations:** 1 Department of Neural and Pain Sciences, University of Maryland, School of Dentistry, Program in Neuroscience, Center to Advance Chronic Pain Research, Baltimore, MD 21201, USA; 2 Centrexion Therapeutics, Baltimore, MD 21202, USA

**Keywords:** capsaicin, pain, nociceptors, TRPV1 receptors

## Abstract

Capsaicin is the pungent ingredient of chili peppers and is approved as a topical treatment of neuropathic pain. The analgesia lasts for several months after a single treatment. Capsaicin selectively activates TRPV1, a Ca^2+^-permeable cationic ion channel that is enriched in the terminals of certain nociceptors. Activation is followed by a prolonged decreased response to noxious stimuli. Interest also exists in the use of injectable capsaicin as a treatment for focal pain conditions, such as arthritis and other musculoskeletal conditions. Recently injection of capsaicin showed therapeutic efficacy in patients with Morton’s neuroma, a painful foot condition associated with compression of one of the digital nerves. The relief of pain was associated with no change in tactile sensibility. Though injection evokes short term pain, the brief systemic exposure and potential to establish long term analgesia without other sensory changes creates an attractive clinical profile. Short-term and long-term effects arise from both functional and structural changes in nociceptive terminals. In this review, we discuss how local administration of capsaicin may induce ablation of nociceptive terminals and the clinical implications.

## 1. Introduction

Anyone who has eaten a hot pepper knows about the pungency of capsaicin. Capsaicin’s pro-nociceptive effects are not confined to the mouth, as this molecule evokes pain in multiple other tissues, including the cornea, skin, joints, and muscles. The discovery of the neural receptor, TRPV1 [1], established the basis for this effect, and represented a major advance in understanding how nociceptors (primary afferents that signal pain) are activated.

The capacity of capsaicin to evoke pain is of value commercially. Of course capsaicin is a staple of many diets and in many cuisines, the capsaicin content is very high [2]. Why capsaicin is such a popular spice remains unclear, given that it essentially evokes a burning sensation in the mouth when eaten. Capsaicin is the active agent in “pepper spray,” a deterrent used for riot control and to ward off assailants [3]. As a repellent, capsaicin is used to discourage intrusions by bears, deer, and other mammals [4]. Bird enthusiasts use capsaicin in their feeders to fend off squirrels, given that the TRPV1 receptor in birds is capsaicin-insensitive [5].

The activation of nociceptors is ordinarily linked with at least the threat of tissue harm. This imposes limits on the use of heat, chemical, and mechanical stimuli to study pain particularly in human studies [6,7,8]. Histological studies of the areas where capsaicin is applied reveal no enduring pathological findings, however. Therefore elicitation of pain without tissue harm makes the use of capsaicin attractive in pain studies.

TRPV1 acts as a transduction channel in nociceptors not only for capsaicin analogues, but also for heat, and acid as well. It has been logical therefore to pursue small molecule antagonists as potential new candidates to treat pain. Unfortunately, antagonists also induce hyperthermia due to the critical contribution of TRPV1 to body temperature regulation. Whether these effects can be avoided ultimately has yet to be seen. Antagonists can also block heat sensibility to the extent that patients may be susceptible to burn injury [9].

Paradoxically, however, capsaicin, and its ultra-potent naturally occurring analogue, resiniferatoxin (RTX), have yet a further property—they act as “excitotoxins”. In other words, these molecules have the capacity to both activate and destroy nociceptive terminals [10]. The selective neurotoxic action of capsaicin was first reported in newborn animals [11], which opened research on capsaicin-sensitive neurons in primary afferents. A single systemic injection of high dose of capsaicin into neonatal rats or mice causes loss of a large proportion of primarily small diameter neurons and unmyelinated afferent fibers. In adult rats, systemic administration of capsaicin at extremely high doses may also induce degeneration of DRG neurons and unmyelinated axons although the extent is less than that in neonatal animals [12].

In a sense, a common observation supports the idea that capsaicin is an excitotoxin. People unaccustomed to eating this spice can tolerate only small amounts. However, a regular habit of eating capsaicin leads to tolerability. Higher and higher doses can be consumed without burning pain. This eventual tolerability reflects the ablative effects of the capsaicin on the nociceptive terminals. This capacity to ablate nociceptive afferents specifically has many implications with regard to therapy.

Perhaps the clearest demonstration of the relative specificity of capsaicin in terms of ablative effects was in an experiment by Simone et al. [13]. Up to 20 µg was injected into the skin in normal human volunteers. Psychophysical testing done on subsequent days revealed a selective loss of heat pain sensibility with sparing of touch sensation. Skin biopsies at the site of injection stained with the pan-axonal marker, PGP 9.5, revealed nearly complete ablation of the C fibers in the epidermis days after the capsaicin injection. Further biopsies weeks after the initial injection revealed restoration of innervation consistent with the regeneration of the afferents. Other studies demonstrated similar findings as discussed below [14,15].

With the demonstration of selective but reversible ablative effects, the stage was set to determine the therapeutic effects of capsaicin administration. Could this pungent spice be used to treat pain?

## 2. Therapeutic Uses of Capsaicin

Low concentrations of topical capsaicin have been available “over the counter” for decades for treatment of pain. Daily application is associated with burning pain and trials have shown varying results in terms of efficacy. The daily application discomfort affects compliance and repeated application over a period of weeks may be necessary to get a therapeutic effect. Given the striking effects of intradermal capsaicin, the idea arose that it might be best to begin with a high dose of topical capsaicin such that the acute pain would be circumscribed in duration, and with the expectation that therapeutic effects would follow within days and last weeks to months. The initial open label report of use of this technique suggested efficacy [16]. Trials with topical 8% capsaicin were conducted subsequently in patients with post-herpetic neuralgia which demonstrated both safety and efficacy, leading to US Food and Drug Administration approval (Qutenza^®^, Acorda Therapeutics, Ardsley, NY, USA). The European Medicines Agency has approved Qutenza^®^ for the more general label, neuropathic pain, based on additional clinical data indicating safety and efficacy in painful diabetic neuropathy, and AIDS related neuropathic pain [17,18].

Pre-clinical data supports additional clinical indications. TRPV1-expressing afferents are known to contribute to spontaneous pain in rodents. Intraplantar injection of capsaicin or RTX attenuates development of guarding behavior following incision of hindpaw skin or carrageenan injection [19,20]. Systemic administration of RTX abolishes spontaneous pain following spinal nerve ligation or complete Freund’s adjuvant (CFA) injection evaluated by conditioned place preference in rats [21,22].

Focal injection of vanilloids (referring to capsaicin and other analogues) also attenuates hyperalgesia in the knee joint. TRPV1 and TRPV1-expressing afferents contribute to mechanical hyperalgesia in knee joints. Pharmacological inhibition or genetic ablation of TRPV1 attenuates arthritis-induced hyperalgesia, such as weight-bearing imbalance, in rodent models [23,24,25]. Intraarticular administration of a TRPV1 antagonist suppressed monosodium iodoacetate (MIA) -induced sensitization of knee joint afferents to mechanical stimuli [26]. This is similar to the situations in other deep tissues such as muscle or visceral organs [27,28,29]. Consistently, intraarticular injection of RTX or capsaicin improves weight-distribution behavior in carrageenan or MIA-induced arthritis in rats and mice [30,31,32]. Therefore, focal injection of capsaicin or RTX can be used to provide relief of mechanical hyperalgesia from deep tissues such as muscle or joints.

Application of capsaicin onto nerve trunks produces a selective and long-lasting increase in the threshold for pain from heat stimuli. This change is confined to the skin region served by the treated nerves [33]. Perineural application of RTX also induces a reduction of inflammation-related thermal hyperalgesia in rats [34,35]. Pre-emptive perineural injection of capsaicin or RTX prevents development of post-incisional pain in rats [20,36]. Local injection of capsaicin into an incision site also has analgesic effects [6,14,15].

Lumbar epidural or intrathecal injection of vanilloids produces long-lasting heat hypoalgesia confined to an area innervated by the cauda equina [37,38,39]. Intrathecal RTX also attenuates inflammatory hyperalgesia [40]. In canines, intrathecal RTX decreases bone cancer-related pain behaviors, and improves functions [41,42], suggesting the promising clinical application of this approach.

Limited data are available from double blind randomized trials regarding the use of injected capsaicin to treat pain [43]. One study has suggested the use of instilled capsaicin to treat post-operative pain [44]. Another condition where this strategy has been pursued relates to Morton’s neuroma, a painful condition that affects the foot. The most common location is between the third and fourth metatarsal bones. A focal swelling of the common digital nerve to the third and fourth toes is evident on imaging studies. Orthotics and other conservative measures often fail in helping the patients. Capsaicin injected into the area of the neuroma significantly relieved pain in comparison to placebo [45]. There were no effects on tactile sensibility consistent with the relatively selective effects on nociceptive afferents. No safety concerns were raised. If supported by further trials this approach has the promise of relieving pain with a single injection. Topical high dose capsaicin typically relieves pain for an average time of five months before re-dosing is necessary [46]. The duration of benefit of injected capsaicin has not yet been determined.

## 3. Functional and Histological Effects

**Figure 1:**
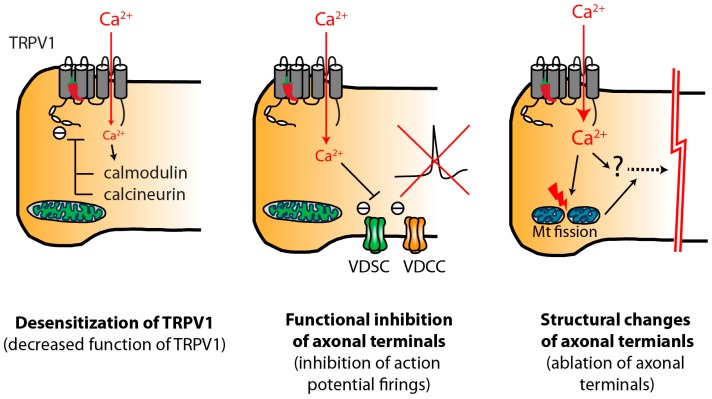
Responses of peripheral terminals of TRPV1-expressing nociceptors following focal injection or topical application of vanilloids. Locally administered capsaicin or RTX induces functional and, potentially, structural changes in nociceptive terminals. With therapeutic doses of capsaicin, these changes are reversible through regenerative mechanisms, and are likely localized to the nerve terminals without affecting the soma. Structural ablation of axonal terminals might play major roles in long-lasting analgesia. TRPV1, transient receptor potential vanilloid subtype 1; VDSC, voltage-dependent sodium channels; VDCC, voltage-dependent calcium channels; Mt, mitochondria.

Focal injection or topical administration of capsaicin activates TRPV1 receptors in TRPV1-expressing nociceptors. This activation is followed by multiple events resulting in functional and potentially histological changes in nerve terminals as summarized in Figure 1.

### 3.1. TRPV1-Expressing Nociceptors

TRPV1, the receptor for capsaicin, is localized primarily in the plasma membrane of Aδ and C fiber primary afferents [1]. TRPV1 is a homo-tetrameric non-selective cationic channel that opens with exposure to agonists. Activation of TRPV1 leads to depolarization associated with the influx of Na^+^ and Ca^2+^ ions. Depolarization is associated with the firing of action potentials in nociceptive fibers which accounts for the capacity of capsaicin to induce burning pain. Since capsaicin-induced nocifensive behavior is ablated in mice lacking TRPV1 expression, capsaicin-induced pain likely depends on activation of TRPV1 [47].

What types of nociceptors signal the pain associated with capsaicin? Different schemes have been used to classify nociceptors and detailed information regarding properties and types of nociceptors has been reviewed elsewhere [48]. One method relates to conduction velocity. Accordingly, there are both Aδ and C fibers. When a heat stimulus is applied to the forearm, there is a double pain sensation. The first, is a sharp pricking sensation and relates to signaling from a type of Aδ nociceptors, and the second is a slow burning sensation, which relates to the discharge of a type of C fiber nociceptors [48,49].

The response to natural stimuli can also be used to classify nociceptors. Nociceptors responsive to heat and mechanical stimuli are referred to AMHs or CMHs depending on whether they are A fibers or C fibers. There are also C and Aδ fibers which are primarily chemically sensitive, and are relatively insensitive to mechanical and heat stimuli. These nociceptors are referred to as CMiHi fibers. As one may infer from the multiple classification systems, nociceptors do not fall neatly into clear discrete categories [48]. Especially confusing is the response to capsaicin. One microneurography study in humans suggested that CMHs account for the magnitude and duration of pain [50]. Further study suggests that capsaicin-induced burning pain in humans is correlated with firings of mechano-insensitive heat-insensitive C fibers (CMiHi) [51]. Intracutaneous injection of capsaicin leads to marked pain during the first 30 s followed by a gradual decrease over the next 5–10 min. Capsaicin induces firing of CMiHi for ~170 s, whereas CMHs discharge only for several seconds, suggesting that capsaicin-induced burning pain maintained for minutes must involve signaling from CMiHi. Furthermore, mechano-insensitive C units become responsive to heat and mechanical stimuli following capsaicin injection, suggesting their role in primary hyperalgesia. Therefore, procedural pain and hyperalgesia following capsaicin injection apparently depends on the sustained discharge of CMiHi units.

A-fiber nociceptors likely also contribute to capsaicin-induced pain. Type I AMHs have a delayed response to heat which increases over time with sustained stimulation. Type II AMHs respond in similar fashion to CMHs and have an immediate response to heat. In primate skin, cutaneous type II AMHs are activated by capsaicin [52]. Upon intradermal injection, the afferent shows strong responses with a high frequency for approximately 15 s, which is followed by low frequency ongoing discharge for approximately 10 min. In contrast, most type I heat-insensitive afferents show only brief high frequency discharge for approximately 5 s without further response. Interestingly, a further subpopulation of heat-insensitive A fiber nociceptors show a vigorous response to capsaicin (>100 action potentials per 10 min).

Nociceptors are also classified based on neurochemical properties. One group expresses neuropeptides such as substance P or calcitonin gene related peptide, and demonstrate a dependency on nerve growth factor. The other class binds isolectin B4 (IB4), and is sensitive to glial cell line-derived neurotrophic factor [53]. TRPV1 is highly enriched in nociceptors containing neuropeptides and approximately 85% of substance P-containing afferents express TRPV1 [54]. IB4-positive neurons also express TRPV1, but to a lesser extent. It is well known that topical application or intradermal injection of capsaicin not only induces burning pain, but also causes flare in human skin [55,56]. Capsaicin induces release and depletion of neuropeptides from afferent terminals, which may lead to attenuation of neurogenic inflammation caused by injury [57]. Capsaicin-induced release of neuropeptides from afferent terminals is primarily due to Ca^2+^ influx through the TRPV1 channel, rather than involving action potential firing since lidocaine, tetrodotoxin, and inhibitors of voltage-gated Ca^2+^ channels do not affect capsaicin-induced release [58,59].

### 3.2. Variations in Acute Pungency of Capsaicin

Injection of capsaicin into peripheral tissues can produce not only spontaneous pain but thermal and mechanical hyperalgesia [60]. In addition to acute pain, injection of capsaicin into the skin induces hyperalgesia to heat stimuli at the site of injection, and stroking pain (allodynia) in the surrounding area. These phenomena again wane within 1–2 h. Hyperalgesia to punctuate stimuli develops in a larger area of skin than thermal or stroking hyperalgesia and lasts up to 24 h. Hyperalgesia that occurs over the skin area outside the injected site is termed secondary hyperalgesia. Secondary hyperalgesia has been determined to result primarily from central sensitization of spinothalamic tract neurons rather than sensitization of the peripheral terminals of nociceptors [61,62].

The above paints the general picture of what happens with acute administration of capsaicin to the skin. However, the extent and duration of acute pain from delivery of capsaicin shows striking variation in humans and animals. This may derive from multiple sources. To begin one has to consider the source of capsaicin. There may be batch to batch variation in the amount of capsaicin and other vanilloids in agriculturally sourced supplies. The formulation used to dissolve capsaicin varies from laboratory to laboratory and this could make a difference. In the case of topical or intradermal delivery, skin temperature can profoundly affect the pain. Where capsaicin is applied on the body clearly matters, though this variable has not received very much attention. Of interest however, is that despite having adequate controls for each of these variables there continues to be considerable inter-individual differences.

In one study polymorphisms of the enzyme, GTP cyclohydrolase, accounted for a surprisingly high (35%) degree of the inter-individual variance in pain ratings from high concentration topical capsaicin [63]. Polymorphisms of catechol-*O*-methyltransferase (COMT) were also associated with nociception following topical application of capsaicin [64]. In another study, other psychological factors were found to account for variations in response to capsaicin [65].

Undoubtedly, a myriad of other factors involved in nociceptive processing will continue to be uncovered. However, one of the particularly compelling variables to consider is the extent to which individual differences in TRPV1 variants account for differences in acute pain. The most common genetic defect in a rare disease known as cystinosis involves a 57k base pair homozygous deletion on chromosome 17, that extends from the cystinosis gene into the early non-coding area for the TRPV1 gene (intron 2). There is a knock down of TRPV1 expression [66] and there was found to be a corresponding decrease in ratings of pain from topical capsaicin [67]. No hyperthermia or inadvertent burns were noted, though there were other possible minor indications of thermoregulatory disturbances and a documented increase in the threshold to warmth stimuli.

Other polymorphisms of TRPV1 are associated with multiple pathological pain conditions such as neuropathic pain, painful osteoarthritis, and dyspepsia. Some genetic variations of TRPV1 occurring in exons alter amino acid sequence of the protein, which affect functional properties of TRPV1 [68,69]. Therefore, genetic variation of TRPV1 may contribute to the variability of pain associated with capsaicin administration [70].

### 3.3. Pungency and Therapeutic Effect?

If capsaicin is to be viewed as an excitotoxin, then one would presume that the “toxic” effects should be correlated with the “excito” effects. As noted above, evidence exists to indicate that injection of capsaicin decreases pain associated with the painful foot condition, Morton’s neuroma. In this study, however, “procedural pain” (acute pain induced by capsaicin administration) was not correlated with therapeutic efficacy [45]. Subjects who reported low levels of procedure pain were just as likely to benefit, and vice versa.

If procedure pain is not a necessary component to the therapeutic effects of capsaicin, then perhaps pungency can be controlled without interfering with analgesic effects. Suppression of procedure pain upon capsaicin injection could also decrease post-procedural discomfort due to mechanical hyperalgesia. One way to cut down on procedural pain is to apply an anesthetic such as lidocaine to the tissue prior to applying capsaicin. Pre-emptive application of lidocaine prior to the application of RTX attenuates acute nociception without affecting analgesic effects in rat cornea [71]. This approach has been tried in an attempt to control the pain associated with Qutenza^®^ application. It is not clear however, that the topically applied lidocaine is of any benefit [72]. A nerve block upstream from the capsaicin blocks all conduction in the nerve and therefore will block all pain from capsaicin applied distally. So why does locally applied lidocaine not have a clear benefit? It is worth noting that lidocaine blocks voltage-gated sodium channels, whereas activation of the TRPV1 channel is associated with an inward current related both to a sodium and calcium ion influx. The failure of lidocaine to be locally effective could relate to the length constant of the sodium current arising from activation of voltage-gated sodium channels relative to the length constant associated with inward current that arises from opening of the TRPV1 channel. If the length constant associated with TRPV1 activation is longer, then the passive current could jump ahead (that is further upstream) and activate voltage-gated sodium channels beyond the point of blockade of the local lidocaine, and thus initiate action potentials that would propagate centrally to produce pain. Though probably not a factor, it is of interest that lidocaine robustly activates TRPV1 over the therapeutic dose range [73].

Interestingly, cold temperature helps to attenuate capsaicin-induced burning pain associated with topical administration [72]. Cold temperature slows down the kinetics of TRPV1 activation by capsaicin [74]. In addition, voltage-gated sodium channels, such as Nav1.7, are largely inactivated at cold temperature, which may reduce the conduction of action potentials [75]. Activation of the cold receptor, TRPM8, could also contribute to analgesia [76]. Despite its benefit, one potential concern is whether maintaining the cold temperature affects the therapeutic effects of capsaicin, since TRPV1 activation by capsaicin is counteracted at temperatures below 15 °C [74]. However, degeneration of epidermal nerve fibers by topical capsaicin was not affected by pretreatment with cooling (20 °C) in humans [72]. As a caveat, more substantial cooling could aggravate pain symptoms by inducing cold hyperalgesia through activation of another nociceptive cold receptor, TRPA1 [77].

Recently, a novel mechanism amplifying capsaicin-induced pungency was suggested. TRPV1 mediated Ca^2+^ influx activates anoctamine 1 (ANO1), Ca^2+^-activated Cl^−^ channels in nociceptive terminals, which leads to further depolarization [78]. Indeed, pharmacological inhibition of ANO1 attenuates capsaicin-mediated acute nocifensive behaviors in rodents [78,79].

### 3.4. Transient Analgesia and Decreased Function of TRPV1 Mechanisms

There are acute effects of capsaicin that could be associated with a component of analgesia, independent of any enduring overt morphological changes. These effects may or may not be of clinical significance. Capsaicin-induced excitation of nociceptors is followed by a refractory state characterized by an insensitivity to subsequent application of capsaicin or other noxious insults such as heat, mechanical or chemical stimuli. To complicate matters, sensitization and desensitization mechanisms are involved since it is observed that topical capsaicin in humans initially decreases heat threshold followed by an increase in threshold [57,80]. The area of skin directly exposed to capsaicin following intradermal injection shows hyposensitivity to pinprick stimuli, which starts as early as 15 min after injection [60]. In rodents, intraplantar injection of RTX immediately induces heat hyperalgesia reflected by a decrease in paw withdrawal latency. This heat hyperalgesia, however, converts to heat hypoalgesia after approximately 2.5 h following injection [34,81]. This quickly developing hypoalgesia may be evident in terms of responses to chemical stimuli and spontaneous pain as well. Formalin-evoked nocifensive behaviors in mice were attenuated by intraplantar injection of capsaicin (100 µg) after two hours. CFA-induced mechanical hyperalgesia was modestly attenuated after 2 h following capsaicin injection (100 µg), which was documented for 24 h [82].

TRPV1 receptor desensitization to vanilloids needs to be distinguished from analgesic effects. The extent of receptor desensitization does not necessarily correlate with the impairment of other nociceptor functions. For example, RTX produces analgesia in vivo but does not induce desensitization of the TRPV1 receptor in in vitro voltage clamp recordings [1]. However, the contribution of TRPV1 receptor desensitization to capsaicin-induced analgesia is unknown. Capsaicin is known to suppress action potential firing in nerve preparations from various species [62,83,84]. In humans, capsaicin suppressed impulse conduction in CMHs, but not cold fibers for example. The conduction block started shortly after application of capsaicin and lasted longer than 2 h [85]. Capsaicin-induced desensitization of nociceptors should involve both Aδ as well as CMH units since Aδ nociceptors mediate first heat pain in humans [49] and topical capsaicin induces heat hypoalgesia mediated by both Aδ and C fiber nociceptors in humans [86]. Indeed, it was shown that subcutaneous injection of RTX attenuates responses of both CMHs and AMHs in rats [87]. Different concentrations of capsaicin result in differential impairment in responses to various stimuli [88]. Dray et al. used a rat spinal cord-tail in vitro preparation to study chemical and thermal stimuli after exposure to low concentration (0.5–2 µM) capsaicin [88], and noted impaired responses to capsaicin but not bradykinin or heat. Function normalized after several hours. In contrast, when the skin was pretreated with a higher concentration of capsaicin (20–50 µM), responses to a broad range of stimuli were impaired irreversibly. These apparently two different types of impairment of responsiveness induced by different concentrations of capsaicin suggest different mechanisms; desensitization of the TRPV1 receptor by low doses of capsaicin versus inhibition of overall nociceptor function by high doses of capsaicin. Exposure of sensory neurons to capsaicin induces ionic currents, whose size decreases during sustained or repeat exposure or by following application of capsaicin. This process is defined as desensitization or tachyphylaxis and is similar to the desensitization that occurs as a result of heat and mechanical stimuli [89,90,91]. TRPV1 is desensitized not only by capsaicin but also heat. The mechanisms are apparently distinct [92]. Capsaicin-induced desensitization of the TRPV1 receptor requires influx of Ca^2+^ through TRPV1, and depends on subsequent Ca^2+^-dependent signaling such as activation of calmodulin and calcineurin, or degradation of PIP_2_ [93]. Capsaicin-induced desensitization of the TRPV1 receptor can be reversible [93]. Desensitization or tachyphylaxis may underlie the selective impairment of the response following exposures to low concentrations of capsaicin.

Studies in dissociated sensory neurons suggest effects of capsaicin on voltage-gated Na^+^ and Ca^2+^ channels. Capsaicin (1 µM) inhibited action potential firing in dissociated sensory neurons from rodents [82,94]. This effect was absent in TRPV1 knockout neurons and depended on Ca^2+^ influx [82]. In capsaicin sensitive rat DRG neurons, a 1 µM concentration inhibited voltage-dependent Na^+^ currents without changing the voltage dependence of activation or markedly changing channel inactivation and use-dependent block [94]. In colon sensory neurons from the rat dorsal root ganglia (DRG), capsaicin inhibited both tetrodotoxin (TTX)-sensitive and TTX-resistant Na^+^ currents. The inhibitory effects were prevented by capsazepine [95] or a specific antagonist of TRPV1, SB366791 [96], suggesting that TRPV1 activation by capsaicin is necessary for the inhibition. Capsaicin also decreased high voltage-activated Ca^2+^ currents through Ca^2+^-dependent calcineurin. This inhibition was prevented by iodoresiniferatoxin, a specific TRPV1 antagonist [97]. Thus several reports support the idea that capsaicin-induced activation of TRPV1 leads to the inhibition of voltage-dependent Na^+^ and Ca^2+^ currents, which in turn suppresses action potential firing in nociceptors. Alternatively, vanilloid-induced membrane reorganization could produce functional suppression of nociceptors. For example, capsaicin and RTX rapidly decrease membrane capacitance of TRPV1-expressing neurons in a Ca^2+^-dependent manner [98]. This effect might involve endocytosis of TRPV1 [99] as well as other ion channels in TRPV1-expressing nociceptive membranes.

Very high doses of capsaicin could potentially affect nociception through effects at the level of the spinal cord [100]. Subcutaneous systemic application of capsaicin at a high dose (20 µmol/kg = 6 mg/kg = 150 µg/mouse) into the scruff of the neck inhibited C-fiber responses in wide dynamic range lumbar dorsal horn neurons activated by transcutaneous electrical stimulation to the hindpaw. The inhibitory effects were suppressed by intrathecal capsazepine [101], suggesting an effect on the central terminals of nociceptive afferents.

Anti-nociceptive or analgesic effects following capsaicin administration might be also derived from the effects of capsaicin on neuropeptide release from primary afferents. Administration of capsaicin decreased substance P from central and peripheral terminals of primary afferents [39,102]. However, the causal relationship between depletion of substance P and capsaicin-induced anti-nociception is unclear [103]. In contrast, capsaicin-induced release of somatostatin, an antinociceptive neuropeptide, could contribute to analgesia. Somatostatin is released from capsaicin-sensitive peptidergic afferents into the circulation and exerts anti-inflammatory and anti-nociceptive effects [104]. An agonist of somatostatin receptor attenuated the responses to formalin, increased the heat threshold, and diminished mechanical allodynia in a diabetic pain model [105]. Carrageenan-induced mechanical hyperalgesia was greater in mice lacking the somatostatin 4 receptor compared to wild-type [106]. Similarly, antinociceptive effects of galanin were also suggested [107]. These reports are consistent with the possibility that capsaicin or RTX administration can induce release of anti-nociceptive peptides, which attenuate hyperalgesia.

The prevailing evidence suggests that capsaicin-induced analgesia mediated by alteration of the functions of nociceptors or release of anti-nociceptive peptides could account for short term effects. Many studies conflate short term and long term effects. Of note, peripheral administration of RTX or capsaicin may induce degeneration of nerve fiber terminals as early as 1 day [13,81]. Therefore, analgesia lasting longer than 1 day may be attributable entirely to the structural ablation. Possibly, acute loss of function following capsaicin injection may be a bodily defense mechanism for reducing acute pungency. A better understanding underlying capsaicin-induced loss of function may help develop methods for reducing capsaicin-induced pungency and procedural pain.

### 3.5. Long Acting Effects of Capsaicin

Focal injection of vanilloids induces long-lasting localized analgesia for weeks to months. Intraplantar injection of RTX was found to induce unilateral hypoalgesia to radiant heat for several weeks in rats [108], likely mediated by effects on Aδ and C-fibers [52]. Intraplantar injection of RTX decreased capsaicin-induced nocifensive behaviors for approximately 40 days and increased latency to hot plate for longer than 60 days in mice [109].

In humans, the topical capsaicin patch (Qutenza®) provides pain relief in post-herpetic neuralgia patients for on average five months [110]. Focal injection of capsaicin in Morton’s neuroma patients provides pain relief for at least four weeks (the longest interval studied) [45]. The likelihood is that analgesia lasting more than a day following localized injection of capsaicin is derived from structural changes. Intradermal injection of capsaicin in humans begins to ablate intraepidermal fibers within one day [13,14]. In rodents, subcutaneous injection of RTX also induced ablation of skin afferent terminals as early as 1–2 days [81,109]. Nerve terminal ablation following local administration of vanilloids is reversible over the time course that correlates with behavioral changes. In mice, TRPV1-positive fibers in skin recover two months following injection of capsaicin [109]. In humans, the number of TRPV1-positive fibers was partially recovered after eight weeks following intradermal capsaicin injection [14]. In another study, regeneration of nerve fibers in humans was demonstrated after 100 days following capsaicin administration [15].

Systemic injection of high doses of RTX or capsaicin degenerates not only peripheral terminals but may also induce substantial ablation of soma in sensory ganglia [12,111]. Systemic injection of >50 mg/kg of capsaicin or >50 ng of RTX are necessary to induce degeneration of ganglia neurons. In contrast, topical application or peripheral injection of a limited dose of capsaicin or RTX ablates nociceptive terminals focally. Local injection of capsaicin or RTX produced ablation of TRPV1-positive afferent terminals in the hindpaw but did not ablate TRPV1-positive afferents in sensory ganglia [108,109]. In humans, therapeutic effects of focally applied capsaicin are reversible, whereas effects on the sensory ganglia are would be expected to be permanent [15]. Intraplantar injection of RTX (~0.5 µg/kg) induced reversible ablation of intraepidermal nerve fibers without degeneration of DRG neurons [108]. In humans, 20 µg of intradermal capsaicin (~0.33 µg/kg) ablated epidermal nerve fibers after 1 day [13]. Of note, at a distance only 1–2 mm from the injection site, the afferents were normal. This makes a more proximal site of action highly unlikely. In Morton’s neuroma patients, pain relief is obtained by injection of 0.1 mg of capsaicin [45], which is almost 30,000 fold lower than the systemic dose for inducing ganglia neuronal degeneration. Therefore, the therapeutic dosage of capsaicin for focal injection is orders of magnitude lower than the dosage resulting in toxicity within sensory ganglia. Even with systemic application, effects may be seen at the level of the peripheral terminals at doses that have no effect on the neurons in the ganglia [112]. In other words, the primary afferent terminals (compared to the soma) are most vulnerable to systemic capsaicin, further evidence that the therapeutic effects of focally applied capsaicin are mediated through local effects.

Although the anti-hyperalgesic effects of perineural application of RTX is apparently reversible [34], perineural application of a high concentration of capsaicin or RTX has been argued to induce a selective but delayed permanent loss of unmyelinated axons and small-diameter DRG neurons or TRPV1-positive DRG neurons [34,113]. It is unclear what mechanisms are involved in transganglionic degeneration following perineural application of capsaicin if indeed it occurs. It is speculated that the extent of axonal injury in a nerve bundle might be great enough to cause a “dying-back” pattern of degeneration, where loss of axonal integrity and transport leads to somatic cell death [114,115]. Regeneration of nociceptor innervation after topical or injected capsaicin argues for intact function at the level of the DRG.

## 4. Potential Mechanisms of Vanilloid-Induced Chemical Ablation of Nociceptor Terminals

The mechanism of vanilloid effects on the nerve terminals may be different than the effects at the ganglion level. Early studies showed that systemic injection of capsaicin to neonatal or adult rat induces irreversible loss of primarily small neurons in sensory ganglia [11]. A single systemic injection of capsaicin into neonatal rats or mice causes a loss of approximately half of the entire DRG neurons and 70%–80% of small diameter DRG neurons. Injection of capsaicin to neonatal rats resulted in losses of ~90% of unmyelinated and ~35% of myelinated fibers from L3 and L4 DRG [116]. In adult rats, high-dose systemic administration of capsaicin also induces degeneration of 17% of small and medium sized DRG neurons, and a 45% decrease in the number of unmyelinated axons in the saphenous nerve [12]. Light and electron microscopy revealed clear degenerative changes of axons and sensory ganglia neurons following the systemic application of capsaicin [12]. Neuronal cell death was suggested to be due to an apoptotic or necrotic mechanism [117,118]. Activation of caspase and DNA fragmentation in DRG neurons following capsaicin administration suggests apoptotic mechanisms [119]. Earlier events following capsaicin administration occurs in cytoplasmic organelles. Dilation of endoplasmic reticulum and swelling of mitochondria was seen following capsaicin treatment [120], which is reminiscent of excitotoxic neuronal death triggered by activation of glutamate receptors [121]. Excessive activation by glutamate mediates death of central neurons through Ca^2+^ overload. Indeed, capsaicin and glutamate both induce accumulation of Ca^2+^ predominantly in mitochondria of the damaged ganglion neurons, suggesting Ca^2+^-dependent neurotoxic effects of capsaicin [122]. In vagal sensory neurons, capsaicin increased permeability to Ca^2+^, and capsaicin-induced ultrastructural changes were attenuated by removing extracellular Ca^2+^ [123]. In dissociated sensory neurons, capsaicin and RTX induced Ca^2+^ uptake in a subpopulation of neurons [124]. Again, capsaicin induced Ca^2+^ entry in dissociated sensory neurons and capsaicin-induced death of DRG neurons was prevented by removing extracellular Ca^2+^ [125]. Of note, heterologous expression of recombinant TRPV1 in non-neuronal cell line confers a liability for capsaicin toxicity [1]. In non-neuronal cell lines with heterologous expression of TRPV1 [126], RTX induced Ca^2+^ influx followed by vesiculation of the mitochondria and the endoplasmic reticulum (∼1 min), nuclear membrane disruption (5–10 min), and cell lysis (1–2 h). RTX also induced Ca^2+^ influx and fragmentation of mitochondria (<20 s) restricted to small size DRG neurons with sparing of glia. In aggregate these reports strongly suggest that capsaicin-mediated neurotoxic effects on sensory ganglia neurons are initiated by Ca^2+^-influx following the activation of TRPV1 in a subset of TRPV1-expressing neurons.

Cytostolic Ca^2+^ can derive also from intracellular sources. TRPV1 receptors are found on the endoplasmic reticulum (ER) [127,128]. Ca^2+^ influx-induced Ca^2+^ release from, in part, the thapsigargin-sensitive Ca^2+^ pool caused cytosolic Ca^2+^ increase [127]. It is not known whether Ca^2+^ released from ER contributes to cell death of sensory neurons following the application of capsaicin or RTX. However, the ER is reported to play a role in cell death of tumor cells through the ER stress pathway involving activation of transcription factor-3 (ATF3) [129] or the mitochondria-mediated death pathway [130]. Since capsaicin induces TRPV1-dependent activation of ATF-3 in sensory ganglia [87,131], it is possible that ER stress following TRPV1 activation might contribute to cell death of sensory neurons.

Intracellular Ca^2+^ homeostasis is critical for physiological functions of nociceptors [97]. In sensory neurons, several mechanisms for controlling Ca^2+^ clearance and homeostasis are known. Ca^2+^ extrusion through plasma membrane Ca^2+^ ATPase, and sequestration of Ca^2+^ into mitochondria, predominantly determine the rate of Ca^2+^ clearance in sensory neurons [132,133]. Increased cytosolic Ca^2+^ mediated by mild electrical stimulation is rapidly cleared by the function of the Ca^2+^ uniporter in the mitochondria membrane, leading to increased mitochondrial Ca^2+^ [134]. However, when Ca^2+^ influx is excessive due for example to intense stimuli, the sequestration capacity by mitochondria proves inadequate, leading to a prolonged elevation of the cytosolic Ca^2+^ level. This happens in sensory neurons following capsaicin application [135].

Excessive Ca^2+^ accumulation in mitochondria is a well-established cause of neuronal excitotoxicity [136]. Ca^2+^ accumulation also leads to the opening of the mitochondrial permeability transition pore (mPTP), a large conductance pore in the mitochondrial membrane that is associated with neuronal apoptosis and necrotic death [137]. Indeed, capsaicin induced death of mesencephalic neurons may result from mitochondrial release of cytochrome c followed by caspase-3 activation leading to apoptosis [138]. Furthermore, pharmacological inhibitors of mPTP attenuated capsaicin-induced death of sensory neurons [139]. Mitochondrial Ca^2+^ accumulation also generates reactive oxygen species (ROS) [140], and mitochondrial Ca^2+^-uptake induces ROS generation in the spinal cord, which contributes to central neuronal plasticity and persistent pain [141,142]. As part of a vicious cycle, mPTP opening is further enhanced by ROS [143]. Clearly, mitochondria could contribute to vanilloid-induced sensory neuronal toxicity due to Ca^2+^ overloading associated with opening of mPTP and ROS generation. Further evidence supports the hypothesis that capsaicin toxic effects are mediated through mitochondrial mechanisms [144]. In sensory neurons, 50 µM capsaicin dissipates the mitochondrial membrane potential as effectively as carbonyl cyanide *p*-trifluoro-methoxyphenylhydrazone (FCCP), a mitochondrial uncoupler. Capsaicin-mediated mitochondrial depolarization is attenuated, but not eliminated, by removing extracellular Ca^2+^. It is not settled, however, whether the effects of capsaicin on mitochondria effects are due entirely to TRPV1-mediated phenomena, and whether these effects fully account for capsaicin toxicity.

Capsaicin-induced cell death is also dependent on calpain. Calpains are Ca^2+^-dependent cysteine proteases associated with multiple neuronal and non-neuronal pathologies [145]. In dissociated DRG neurons, calpain inhibitors attenuated capsaicin-induced cell death independent of a capsaicin-induced cytosolic Ca^2+^ increase [125]. Capsaicin increased breakdown products of α-spectrin, a cytoskeletal target of calpain, which was prevented by a calpain inhibitor. These results suggest that capsaicin-induced activation of calpains contributes to cytotoxicity by perturbing the cytoskeleton. Alternatively, calpain activation may lead to apoptosis. In a breast cell line, capsaicin-induced ER stress elevated intracellular Ca^2+^ leading in turn to calpain activation, and apoptosis related to mitochondrial effects [130].

Since capsaicin-induced cell death involves multiple Ca^2+^-dependent processes, it is likely that these contributors may also mediate capsaicin-induced ablation of axonal terminals. Studies of mitochondrial fission support the role of mitochondria [146]. In dissociated DRG neurons, capsaicin induced axonal swelling, which was accompanied by reduction in the length of the mitochondria and motility within axons. These changes in mitochondria were attenuated by Ca^2+^ chelators and capsazepine. Transfection with a dominant negative mutant Drp1, a mitochondrial protein responsible for mitochondrial fission, attenuated the capsaicin-induced decrease in mitochondrial length as well as axonal swelling and degeneration. Prevention of mitochondrial fission also prevented capsaicin-induced loss of the mitochondrial membrane potential. Overall this study strongly supports the contribution of mitochondrial mechanisms as an explanation of capsaicin effects.

## 5. Clinical Correlates

The capacity of capsaicin to ablate nociceptive terminals that express TRPV1 has many therapeutic implications. The agonist effects of capsaicin are not to be confused with the effects of TRPV1 antagonism. A putative TRPV1 antagonist in principle works only on TRPV1 mediated transduction, leaving intact other transduction mechanisms. Capsaicin, as a TRPV1 *agonist*, and as an excitotoxin, by ablating the terminals of nociceptors blocks other transduction mechanisms that may be co-expressed in the nociceptors. For example, TRPA1 is co-expressed with TRPV1 [147]. Thus capsaicin would be expected to block TRPA1 to the extent that TRPA1 is co-expressed with TRPV1. This expands the therapeutic window of an agonist, such as capsaicin, in terms of long term therapeutic effects. One injection is expected to be analgesic for weeks to months, despite a half-life in the blood that lasts for minutes to hours. The additional upside is that all of the functions of the nociceptor are affected. The TRPV1 receptor, in some ways, is simply the Trojan horse.

Capsaicin effects are highly localized to the area of injection. In work of Simone et al. [13], at a distance of only 1–2 mm from the site of intradermal injection, no effects of the capsaicin were seen. Therefore, a pain problem that is widespread would be impractical to treat with topical or injected capsaicin. Another consideration is that TRPV1 is not expressed in all nociceptors. The efficacy of capsaicin will depend on the extent to which the signaling nociceptors that produce the pain express TRPV1. To that point, the predominant long term sensory effect of injected capsaicin in the skin is on heat sensibility with a much lesser effect on pain from mechanical stimuli [13]. Other evidence indicates that mechanical pain in muscle does involve TRPV1 [28,29].

Another important consideration regards the involvement of peripheral and central mechanisms. This is best understood with injury to the skin. Treede and colleagues [61] used electrical stimuli to induce local pain, allodynia, and hyperalgesia. High concentration topical capsaicin (8%) was used to knock out innervation of nociceptors that expressed TRPV1. Pain to the electrical stimuli itself was dropped by about half. The secondary hyperalgesia and the allodynia, both likely due to central mechanisms (central sensitization), were almost entirely eliminated. Secondary hyperalgesia is characterized by heightened mechanical pain (not heat) and results from the initial sensitization of nociceptors at the point of injury. Blocking the input of nociceptors at the point of injury probably accounts for the elimination of abnormal mechanical pain in the secondary zone. This means that capsaicin may affect abnormal mechanical pain indirectly by blocking central sensitization via effects on the primary afferents and peripheral sensitization [61]. Given the likely importance of central mechanisms in most clinical pain problems, these data suggest that a knockout of TRPV1 expressing nociceptors by capsaicin has favorable prospects as a useful therapy. Likewise, the therapeutic application of RTX targeting TRPV1 expressing nociceptors has been advocated [148].

Further support relates to the evidence that indicates up-regulation of the expression and function of TRPV1 receptors in different disease states such as cancer, visceral inflammation, and neuropathic pain [27,149,150,151,152,153]. Therefore, the role of TRPV1 in nociception may evolve to be of greater importance in disease. Heat sensitization may be argued to have little importance with regard to deeper tissues. However, sensitization to the point that nociceptors are activated by the ambient core temperature or locally produced endogenous ligands, indicates a means by which TRPV1 bearing nociceptors may play an pivotal role in “ongoing” or spontaneous pain associated with clinical pain states.

Beneficial effects of ablation of nociceptive terminals are likely maintained for weeks to months, as regeneration of nociceptive fibers reestablishes innervation of the target tissues. Patients might be reinjected in order to extend the therapeutic effects. The mechanisms underlying regeneration following capsaicin-induced ablation may be similar to the regeneration mechanisms that follow axotomy. A subpopulation of skin nerve fibers showed immunoreactivity to GAP43, a marker of regenerating nerves, following topical administration of capsaicin in humans, suggesting regeneration of primary afferents [14]. The intraplantar injection of RTX increases ATF3 and galanin, markers of nerve regeneration, within DRG for up to 10 days suggesting that regeneration processes follow terminal ablation [87]. Since the duration of therapeutic effects of vanilloid-induced analgesia may be correlated with the time course associated with regeneration, better understanding of the mechanisms of regeneration could help to determine ways to extend the duration of effects.

## 6. Conclusions

The cloning of the TRPV1 receptor ignited a new era of pain research by opening the gateway to understanding how nociceptors are activated. In this same timeframe the study of ligands, such as capsaicin and RTX, also intensified. Capsaicin was recognized as a molecule that could be used as an experimental stimulus to evoke pain. Appreciation also grew that capsaicin was not only powerfully algesic, but that it also led to focal degeneration of nociceptors. High concentration topical capsaicin was approved to treat post-herpetic neuralgia and, in Europe, other neuropathic pain conditions. In focal pain conditions there is appreciation that capsaicin may be given by injection to knockout nociceptors and achieve pain control. Studies in Morton’s neuroma, a painful neuropathic pain condition, have shown promise. Injection may also be useful for direct delivery into a joint to control arthritis pain. Other uses for many other pain conditions may evolve.

The major impediment to the clinical use of capsaicin is the immediate pungency. However, with the high dosing associated with topical use or injection to treat Morton’s neuroma, for example, the immediate application pain is quite circumscribed, lasting in the order of minutes to hours in exchange for months of therapeutic benefit.

A future challenge for TRPV1 excitotoxins is to mitigate the “excite”, and accentuate the “toxin” aspect of the effects. Further understanding the cellular mechanisms of action may very well help fulfill this promise.
